# Minimum size and positioning of imaging field for CBCT-scans of impacted lower third molars: a retrospective study

**DOI:** 10.1186/s12903-021-02029-6

**Published:** 2021-12-29

**Authors:** Anne-Mari Ilo, Marja Ekholm, Elmira Pakbaznejad Esmaeili, Janna Waltimo-Sirén

**Affiliations:** 1 Department of Oral Pathology and Oral Radiology, Institute of Dentistry, University of Turku, Lemminkäisenkatu 2, 20014 Turku, Finland; 2 Department of Oral and Maxillofacial Diseases, University of Helsinki, Helsinki, Finland; 3 South West Finland Imaging Centre, Turku University Hospital, Turku, Finland; 4 Department of Pediatric Dentistry and Orthodontics, Institute of Dentistry, University of Turku, Turku, Finland; 5 City of Turku, Division of Welfare, Turku, Finland; 6 Department of Oral and Maxillofacial Diseases, Helsinki University Hospital, Helsinki, Finland

**Keywords:** Cone-beam computed tomography, Indication-specific imaging field, Third molar imaging, Indication-specific field-of-view placement

## Abstract

**Background:**

Cone-beam Computed Tomography (CBCT) is widely used for preoperative 3D imaging of lower third molars. Hence, for this imaging indication, the present study aimed to define the minimum field-of-view (FOV) size and its optimum placement, to decrease radiation exposure, and highlight the need of computer-assisted FOV centering technique for dental CBCT devices. To facilitate proper placement of image field, lower second molar was chosen as reference.

**Methods:**

The retrospective study included 50 CBCT-scans of 46 patients with mean age of 34 years. Based on the lower second molar, a three-dimensional coordinate was formed and the location of mandibular canal (MC) and the dimensions and locations of the lower third molars, and possible associated pathological findings were assessed. Accordingly, the FOV size and position for third-molar imaging were optimized, while ensuring encompassment of all relevant structures.

**Results:**

The minimum cylindrical volume, covering lower third molars and MC, was 32.1 (diameter) × 31.6 (height) mm, placed in relation to the second molar crown, top 2.2 mm above cusp tips, anterior edge 6.7 mm in the front of the most distal point of the crown, and lingual edge 7.9 mm on the medial side of the lingual wall.

**Conclusions:**

The optimized FOV for lower third molars was smaller than common standard small FOVs. We recommend using FOV volume 3.5∅ × 3.5 cm for third molars without associated pathology. Accurate FOV protocols are essential for development of new CBCT-devices with computer-assisted and indication-specific FOV placement.

## Background

Third molar extraction is one of the most common procedures in maxillofacial surgery [1] with 10 million third molars extracted from 5 million people in the United States each year [2]. Preoperatively, radiographic examination is needed to assess the root morphology of the third molar and the relationship between the tooth, the adjacent second molar, and the mandibular canal (MC). Damaging MC structures, particularly the inferior alveolar nerve, may cause postoperative complications, such as neurosensory deficits [1, 3–5]. The primary imaging method for third molars is dental panoramic tomography (DPT) [6]. DPT gives a good two-dimensional (2D) overview of the teeth and jaws, but regarding proximity of different anatomical structures or root resorption it does not facilitate reliable assessment. Cone-beam computed tomography (CBCT) has been gaining popularity due to enabling three-dimensional (3D) visualization of teeth, jaws and related bony structures [5, 7, 8]. A Finnish nationwide survey reported that of the monthly 1,345 CBCT-scans 25% were due to mandibular third molars, making the assessment of the relationship between the lower third molar and MC the third most common clinical indication for CBCT-imaging [9].

Rapid development of CBCT-technology and access to CBCT-devices have increased the associated risk from radiation exposure received by patient because radiation doses are generally higher in CBCT than in conventional radiography [7, 10–13]. Majority of third molars are extracted from 20- to 30-year-old patients who are more vulnerable to ionizing radiation than older patients [7, 14]. To improve radiation safety in CBCT examination, the imaging parameters should be selected according to the diagnostic task and patient-specific indications and optimized according to ALARA principle (As Low As Reasonably Achievable) [7, 13, 15]. CBCT guidelines in the North America conclude that the selected field-of-view (FOV) is an important consideration to reduce to dose to the patient [16]. The size and positioning of FOV affect the effective radiation dose [8, 10, 11, 17]. Using small FOV (< 10 cm height), compared to large FOV sizes (> 15 cm height), the mean effective doses could decrease by 60% [18]. The general guideline is to keep FOV as small as possible, encompassing only the region of interest [7, 8, 11].

Studies about optimization of exposure parameters in standard FOVs exist, but exact indication-specific imaging protocols with FOV adjustment are rare in the literature [4, 13, 15]. Recommendations on FOV size and optimal positioning have been published for imaging of impacted maxillary canines [19]. Defining the optimum FOV is a major requirement for indication-specific optimization with advanced CBCT-technology. Automatic FOV centering and patient positioning improves radiation dose optimization in medical computed tomography (CT) examinations [20, 21], and such technology is similarly desirable in the dental field.

Accordingly, the aim of this retrospective observational study was to find the smallest diagnostic FOV size for impacted or partially erupted lower third molars and defining its adequate positioning.

## Methods

The study follows STROBE guidelines for reporting observational studies [22]. Spatial location of the third molar in relation to second molar coronal landmarks forms the primary outcome and delineates the optimized FOV in terms of its size and placement, which are the secondary outcomes of this research.

The data collection contained 127 consecutive CBCT-scans of lower third molar, performed at Department of Oral and Maxillofacial Diseases, University of Helsinki, Finland, between August 2014 and September 2015. In terms of the assessment of lower third molar and MC, all CBCT-scans were diagnostic with the sufficient FOV. Due to the present study protocol where the lower second molar served as a reference, 77 CBCT-scans were excluded from further analysis since the lower second molar was not fully visible in the scan volume (n = 70), it was infra-occlusal (n = 3), or it was extensively restored (n = 4). The FOV sizes of the finally included 50 CBCT-scans were 4 × 5 cm (n = 36) or 8 × 5 cm (n = 14) and voxel sizes 200 µm (n = 31) and 150 µm (n = 19) had been used.

The CBCT-scans were performed with Promax 3D (Planmeca, Helsinki, Finland). All radiographic data were viewed in a room with optimal ambient lighting conditions and using Romexis®-software (Planmeca, Helsinki, Finland). The data were analyzed, and measurements made by one examiner (A-M.I.). Unclear cases were reviewed with a senior oral radiologist (M.E.).

### Size and position of a cylindrical volume

In CBCT, FOV is a cylindrical volume reported as D∅ x H cm, where D is the diameter and H is the height. To define minimum size and position of the cylindrical volume, accurate 3D localization of lower third molar, the MC, and eventual pathological findings were measured in horizontal, frontal and sagittal planes. Analysis comprised three steps: i) setting a 3D coordinate system, based on and including the second molar, ii) defining the position of each of the 50 third molars, the MC and eventual pathological findings in relation to the coordinate, and iii) combining the data to delineate an optimized CBCT-volume and its positioning.

#### 3D coordinate

**Fig. 1:**
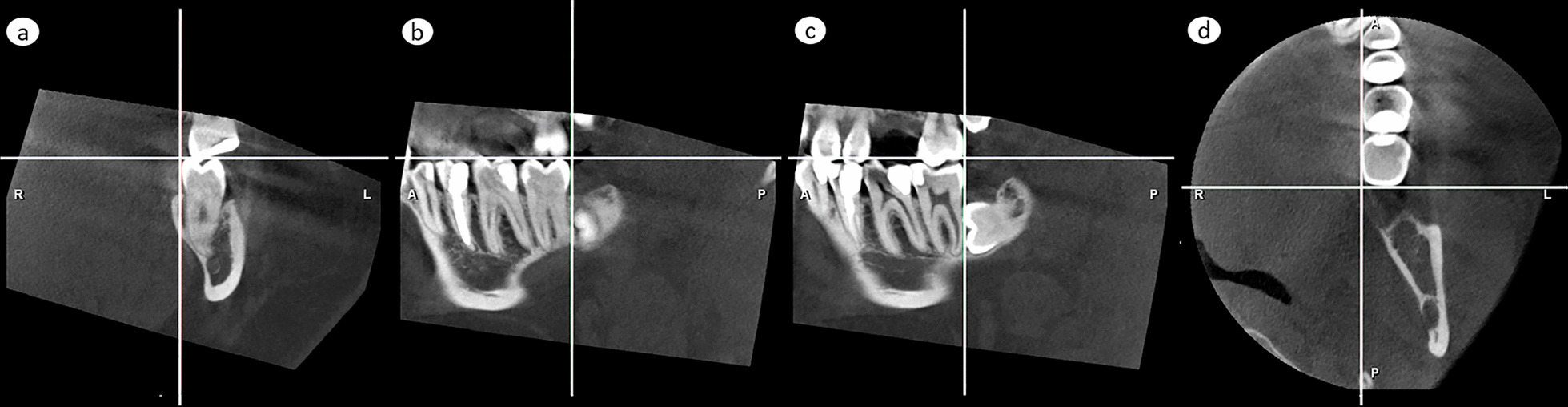
Formation of the X–Y–Z coordinated three reference planes, perpendicular to each other and based on the lower second molar structures. **a**, **b** Horizontal plane, illustrated by the horizontal line, was set to tangent the disto-lingual and disto-buccal cusp tips, as seen in a coronal view in (**a**), and at the same time also the mesio-lingual cusp tip, as seen in sagittal view in (**b**). **c**, **d**. Frontal plane, set to touch the most distal point of the crown, is shown as a vertical line in a sagittal view in (**c**) and as a horizontal line in an axial view in (**d**). **a**, **d** Sagittal plane, set to touch the lingual wall of the crown, is shown by the vertical lines in a frontal view in (**a**) and in an axial view in (**d**)

An impacted lower third molar is not clinically visible, and to facilitate the proper placement of the CBCT-volume, the lower second molar was chosen as reference. To place the lower second molar in a constant position in X-Y-Z-coordinate, three anatomical planes in perpendicular relation to each other were set as reference planes, using anatomical landmarks of the lower second molar as follows:

1. Horizontal plane; set according to disto-lingual and disto-buccal cusp tips of the lower second molar in a coronal view (Fig. 1a), and at the same time according to its mesio-lingual and disto-lingual cusp tips in a sagittal view (Fig. 1b)
2. Frontal plane; set at the most distal point of the lower second molar crown in sagittal view (Fig. 1c) and in axial view (Fig. 1d)
3. Sagittal plane; set along the most prominent part of lingual wall of the lower second molar crown in coronal view (Fig. 1a) and axial view (Fig. 1d)

#### Position of lower third molar, the MC and pathological findings

**Fig. 2:**
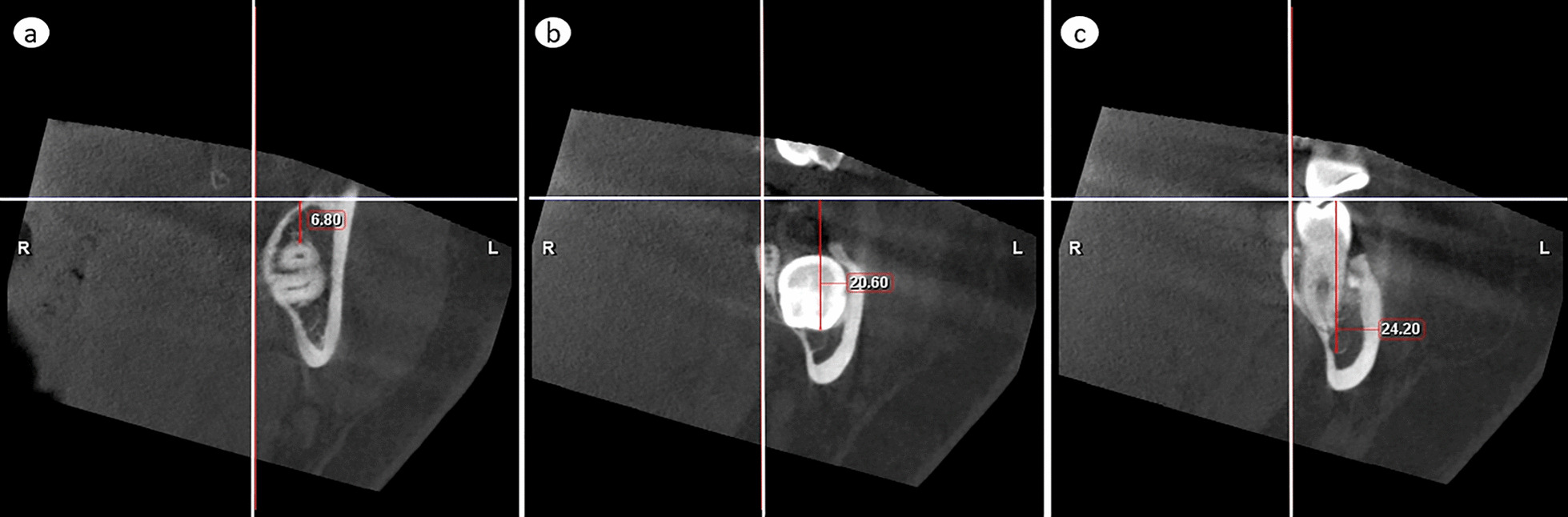
Vertical measurements. **a** The most superior and **b** the most inferior distance of any part of the lower third molar from the horizontal plane. **c** The most inferior distance of the inferior cortical border of the mandibular canal from the horizontal plane
  2. most inferior part from the horizontal plane (Fig. 2b)
  3. most anterior part from the frontal plane (Fig. 3a)

To measure the dimensions of the third molar and to localize lower third molar, the MC, and eventual pathological findings with the second molar as reference, the extreme distances in both directions were measured separately in relation to each of the above given three reference planes. In horizontal direction, the most superior distances from the horizontal plane delineated top of the cylinder and the most inferior distances bottom of the cylinder. Combination of horizontal measurements defined height and location of the cylinder in cranio-caudal direction. In frontal direction, the most anterior distances delineated anterior edge of the cylinder and the most posterior distances posterior edge of the cylinder. In sagittal direction, the most medial distances delineated medial edge of the cylinder and the most lateral distances lateral edge of the cylinder. Combination of the frontal and sagittal measurements delineated the diameter of the cylinder and defined its location in anterior–posterior and medio-lateral directions. These distances were measured as follows:

- Distance of the lower third molar’s
  1. most superior part from the horizontal plane (Fig. 2a)

**Fig. 3:**
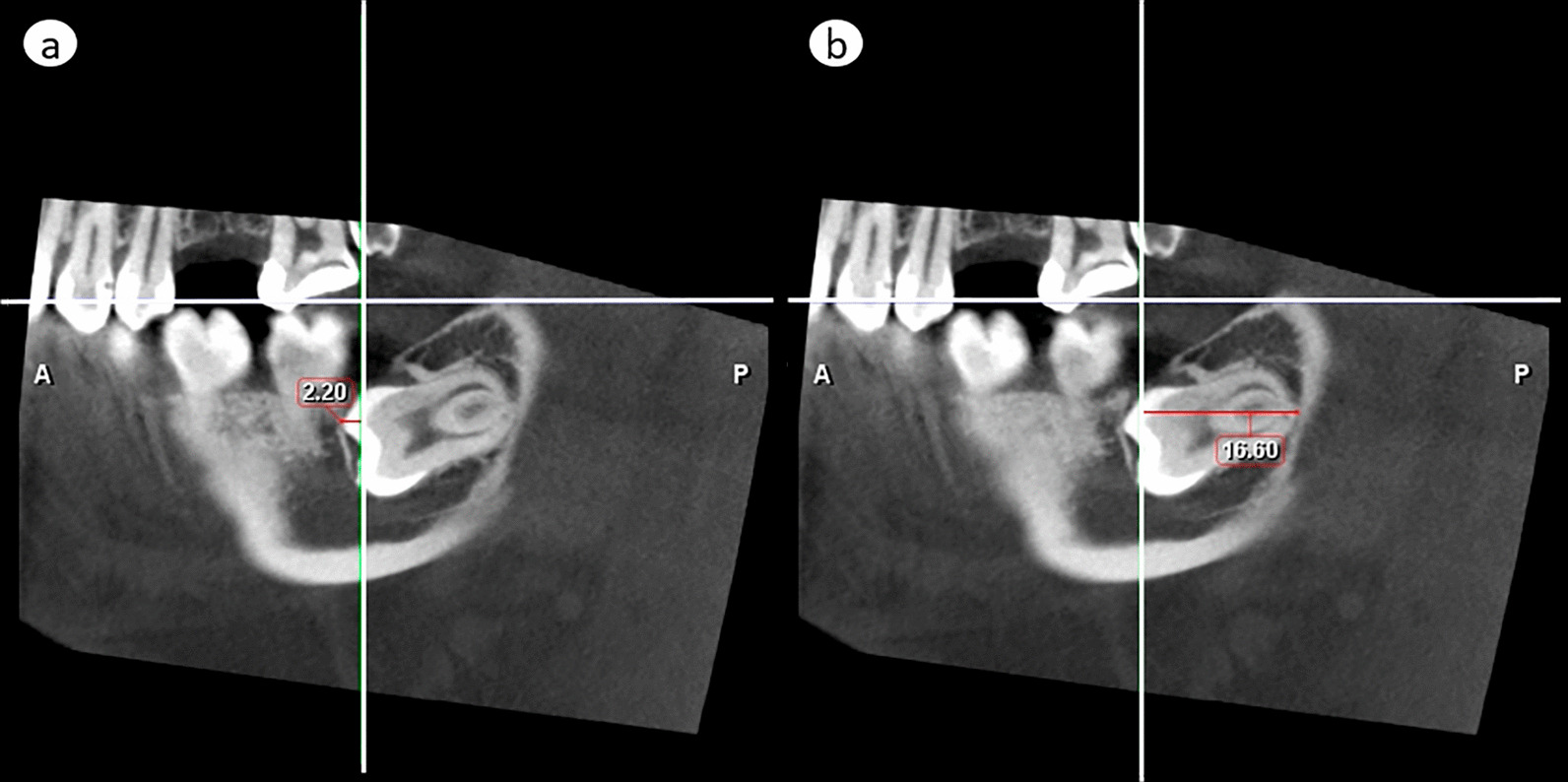
Antero-posterior measurements. **a** The most anterior and **b** the most posterior distance of any part of the lower third molar from the frontal plane. If the tooth crosses to the anterior side of the frontal reference line, like in (**a**), the value is negative
  4. most posterior part from the frontal plane (Fig. 3b)
  5. most medial part from the sagittal plane (Fig. 4a)

**Fig. 4:**
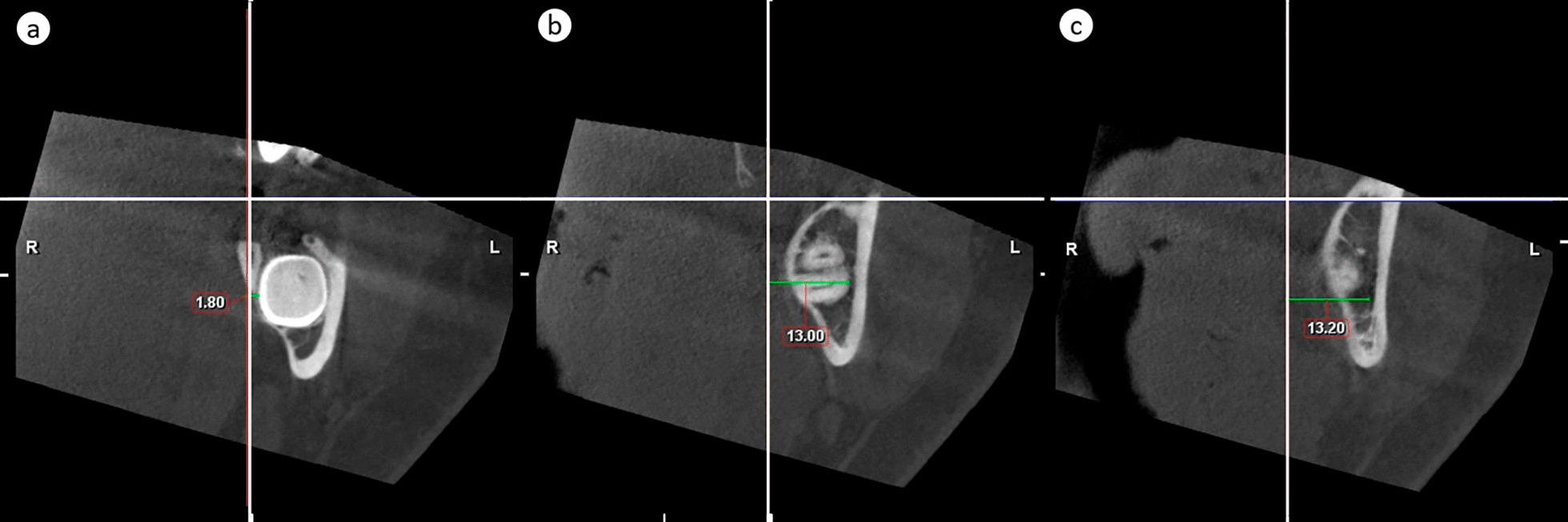
Medio-lateral measurements. **a** The most medial and **b** the most lateral distance of any part of the lower third molar from the sagittal plane. **c** The most lateral distance of the lateral cortical border of the mandibular canal from the sagittal plane
  6. most lateral part from the sagittal plane (Fig. 4b)
- Distance of the cortical borders of MC in the region of the lower third molar
  1. most inferior point from the horizontal plane (Fig. 2c)
  2. most lateral point from the sagittal plane (Fig. 4c)
- Distance of pathological findings associated with lower third molar (width of follicular space > 3 mm) reported similar to those in a).

#### Data combination

The information obtained from the individual scans were combined.

- to define the dimensions of the smallest 3D size of a cylinder that totally encompasses all impacted or partially erupted lower third molars as well as the MC. Data for right and left sides were analyzed separately. Third molars with pathological findings were evaluated separately.
- to define the position of the assessed cylinders, in order to optimize their placement.

### Root development

As our aim was to define minimum FOV size that would be sufficient to cover third molars of any developmental stage, we evaluated developmental stage of all third molars to ensure that our data contains sufficient number of teeth of full root length development. Developmental stage was classified according to Liversidge [23]. The classification defines 15 tooth-developmental stages from crypt to two last stages concerning the development of the apex: At stage A½, the apex of distal root is partially open and periodontal ligament is still slightly wider, while at the final stage Ac, the apex is closed, and the periodontal ligament displays uniform width [23].

### Eruption and inclination

The stage of eruption of the lower third molar was classified in relation to the marginal bone cortex:

1. The crown perforates the marginal bone cortex
2. The crown is in contact with the marginal bone cortex
3. The entire crown is subcortical

The inclination of the lower third molar was measured by an angle between the long axes of the lower second and third molars. We used the previously defined frontal plane as the long axis of the lower second molar. The long axis of the lower third molar was defined as a line drawn perpendicular to a line marking the maximum mesiodistal third-molar crown width. The lower third molars were categorized into four groups based on inclination classification by Singh et al. [24]:

- Vertical, ± 10°
- Mesioangular, + (11°–70°)
- Distoangular, − (11°–70°)
- Horizontal, >  ± 71°

### Intra-examiner reproducibility and statistical analysis

Reliability of analyses was tested by evaluating the reproducibility of inclination and tooth positioning measurements. Tooth inclination was reassessed after 6 months on 15 randomly picked scans. After 12 months, a new random sample of 15 scans was picked and the original settings were restored, all reference planes were set again, and position measurements were repeated. Intra-examiner reproducibility was analyzed by calculating the range of error, mean error, and random error with 95% confidence interval, using the formula of the ‘method of moment’ estimator (MME) [25]. Two-tailed t-test was performed with Microsoft Excel (Microsoft Corporation, Redmond, WA, USA).

## Results

The 50 CBCT-scans were of 46 patients, 32 females and 14 males, aged 19 to 67 years with a mean age of 34. Of the 50 lower third molars, 23 were on the right side. All 50 teeth had perforated the marginal bone cortex. Categorization by inclination: vertical (n = 16), distoangular (n = 15), mesioangular (n = 10) and horizontal (n = 9), and the subgroup with pathological findings were: distoangular (n = 5), mesioangular (n = 4), vertical (n = 3) and horizontal (n = 2). Developmental stages of 49 lower third molars were stage Ac and one represented stage Rc (root completed).

### Pathological findings

Pathological findings associated with the lower third molars were detected in 14 scans: 7 with pericoronitis and 7 with suspicion of dentigerous cyst. One dentigerous-cyst suspicion and 4 pericoronitides (n = 5) were adjacent to the right-side lower third molar, while 6 dentigerous-cyst suspicions and 3 pericoronitides (n = 9) to the left.

### Location of lower third molars and mandibular canal

#### Cranio-caudal assessment

**Fig. 5:**
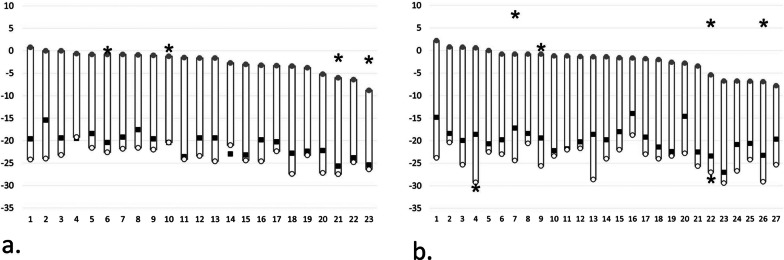
Vertical distances (mm) of the **a** right-side and **b** left-side lower third molars (filled circle the most superior and filled square the most inferior part), associated pathological findings (asterisk the most superior and inferior parts), and mandibular canal cortex (circle the most inferior part) in relation to the horizontal reference plane (level 0; adjusted by the disto-lingual, disto-buccal and mesio-lingual cusps of the lower second molar). Out of all measured values of the pathological findings, only those that increase vertical height of the cylinder are shown in the diagram

The most superior part of any right-side lower third molar was located at maximum 0.8 mm above the horizontal plane, and the most inferior point at maximum 25.7 mm below it. The inferior border of the MC cortex was located, at most, 27.5 mm below the level of the horizontal plane (Fig. 5a). On the left, the most superior part of any lower third molar was located at maximum 2.2 mm above the horizontal plane, and the most inferior point at maximum 27.0 mm below it. The inferior border of the MC cortex was located, at most, 29.4 mm below the level of the horizontal plane (Fig. 5b).

#### Antero-posterior assessment

**Fig. 6:**
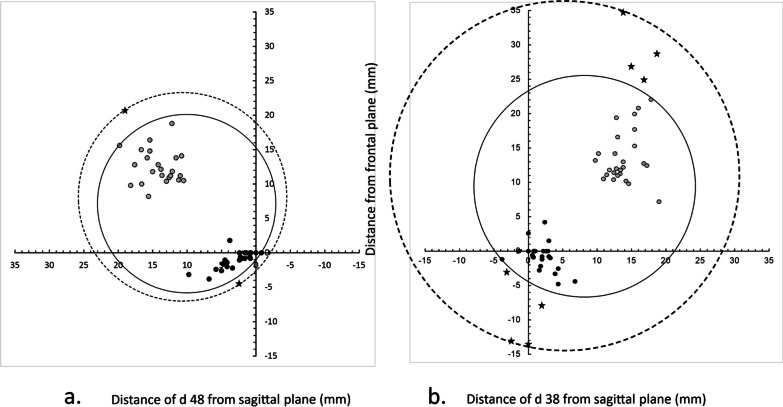
Horizontal positions of the 23 right-side and 27 left-side lower third molars. Their minimum and maximum medio-lateral (x-axis) and antero-posterior (y-axis) distances are depicted two-dimensionally from the reference planes (levels 0), the sagittal plane set according to the lingual prominence point and the frontal plane according to the distal prominence point of the lower second-molar crown. The circles illustrate the optimized diameter and horizontal positioning of vertical cylinders that fully encompass these lower third molars and the MC. Continuous line—teeth without pathological findings. Dashed line—teeth with pathological findings

The most anterior part of any of the right-side lower third molars crossed the frontal plane by 3.8 mm, and the most posterior part was at maximum 18.8 mm behind it (Fig. 6a). The most anterior part of any of the left-side lower third molars crossed the frontal plane by 4.8 mm, and the most posterior part was at maximum 22.1 mm behind it (Fig. 6b).

#### Medio-lateral assessment

The most medial part of any of the right-side lower third molars crossed the sagittal plane by 0.8 mm, and the most lateral one was located at furthest 19.8 mm from it (Fig. 6a). The lateral border of the MC cortex was located, at most, 18.4 mm from the sagittal plane. On the left, the most medial part of any lower third molar crossed the sagittal plane by 3.8 mm, and the most lateral one was located at furthest 19.0 mm from it (Fig. 6b). The lateral border of the MC cortex was located, at most, 18.6 mm from the sagittal plane.

### Size of FOV

- without pathological findings

To encompass all right-side lower third molars in any inclination as well as the MC, the required size of a cylinder was 25.9 mm in diameter and 28.3 mm in height. Respectively, the cylinder size for the left side would be 32.1∅ × 31.6 mm (Figs. 5 and 6).

- with pathological findings

The need of encompassment of pathological findings, too, increased the required cylinder size on the right up to 30.5∅ × 28.3 mm, and on the left up to 51.0∅ × 38.6 mm (Figs. 5 and 6).

### Position of FOV

- without pathological findings

To ensure accommodating all right-side lower third molars, and the MC in the 25.9∅ × 28.3 mm volume, the top of the cylinder should be placed 0.8 mm above the horizontal plane, its anterior edge 5.8 mm crossing the frontal plane, and medial edge 2.8 mm crossing the sagittal plane. The adequate positioning on the left side volume, 32.1∅ × 31.6 mm, would be: top 2.2 mm above the horizontal plane, anterior edge 6.7 mm on the anterior side of the frontal plane, and medial edge 7.9 mm on the medial side of the sagittal plane (Figs. 5 and 6).

- with pathological findings

The optimal positioning of the cylindrical 30.5∅ × 28.3 mm volume that additionally encompasses the pathological findings would be on the right side: top 0.8 mm above the horizontal plane, anterior edge 7.0 mm anterior to the frontal plane, and medial edge 4.5 mm crossing the sagittal plane. On the left side, top of the 51.0∅ × 38.6 mm volume would be 8.0 mm above the horizontal plane, anterior edge 14.6 mm anterior to the frontal plane, and medial edge 20.2 mm crossing the sagittal plane (Figs. 5 and 6).

### Combined results for right- and left-side lower third molars

We did not observe difference between measurements of tooth position between right- and left-side lower third molars (differences between group-mean values ranging from 0.14 mm to 1.21 mm, (0.07 ≤ p ≤ 0.85). Combined results for an adequate cylindrical volume and its positioning are equal to those assessed above for the left side (both including and excluding pathological findings). The resultant positioning of this cylinder covers up to 6.7 mm of the distal part of the second molar, and in case of pathological findings, up to 14.6 mm of it, hence covering the entire second molar.

### Intra-examiner reproducibility

The repeatability of measurements in the three planes observed no difference (0.08 ≤ p ≤ 0.98). Differences between distance measurements varied between 0.00 mm and 2.00 mm and the mean random errors between 0.29 mm and 0.61 mm. The random error of the inclination degree was 2.08°, with a 95% confidence interval of 1.05°-3.12° (Table 1).

**Table 1:** Intra-observer reproducibility of the linear and angular measurements from 15 CBCT-scans

Difference between repeated measurements	Distance of third molar and mandibular canal (MC) from the cranio-caudal reference plane (mm)			Distance of third molar from the antero-posterior reference plane (mm)		Distance of third molar and mandibular canal (MC) from the medio-lateral reference plane (mm)			Degree of inclination (°)
	Minimum	Maximum	MC, maximum	Minimum	Maximum	Minimum	Maximum	MC, maximum	
Range	0.00–1.40	0.00–1.20	0.00–2.00	0.00–1.95	0.00–1.20	0.00–0.80	0.00–1.80	0.00–1.80	0.00–6.31
Mean	0.11	0.11	0.34	0.02	0.11	0.01	0.00	0.14	1.11
Random error	0.35	0.35	0.35	0.45	0.30	0.29	0.45	0.61	2.08
95% CI^a^ of random error	0.18–0.52	0.18–0.52	0.18–0.52	0.23–0.67	0.15–0.45	0.15–0.44	0.23–0.68	0.31–0.92	1.05–3.12

^a^Confidence interval

## Discussion

The present study was carried out to define a minimum FOV size and optimal positioning for CBCT-imaging of lower third molars. The need for guidelines of indication-specific optimization for CBCT-scans has been emphasized in previous studies [13, 15, 19], but, to our best knowledge, minimum FOV size with adequate positioning protocol based on exact 3D location of a tooth has been defined only for impacted maxillary canines [19]. In the present study, the minimum FOV and optimum positioning for imaging of lower third molar were defined according to exact 3D locations of 50 third molar teeth, showing a representative variety of vertical, horizontal, distoangular and mesioangular inclinations and full length of root development. Minimum FOV for lower third molars with pathological findings was defined separately, as the presence of pathological lesions associated with the third molars can be assessed from a precluding DPT, and CBCT-protocol chosen accordingly. Equally, a precluding DPT would reveal an ectopically located third molar and implicate a need for FOV adjustment. Ectopic mandibular third molars, locating any place between the mandibular body and condyle, are rare [26], and were not observed within the present study material.

Exposure parameters, voxel size, scanning technique, and imaging field have an influence on the radiation dose. In addition to FOV size, the position of the FOV has direct impact on effective dose of the patient, depending on the coverage and proximity of radiosensitive organs [8, 17, 27]. A variation in the location of a small FOV (7.5 cm × 10.0 cm) with a constant dose area product (DAP) can cause nearly three-fold change in the effective dose [27]. Mainly due to the vicinity of the thyroid and salivary glands, placing a 7.8 × 15 cm FOV with constant DAP in the mandible region versus maxilla region may double the effective dose [17]. In terms of CBCT-scan optimization for lower third molars, these observations emphasize the relevance of minimum FOV size with adequate positioning.

Common standard small FOV sizes are 4 × 4 cm or 5 × 5 cm [10]. Previous studies on indication-specific FOV-sizes have recorded that small standard FOVs, or even smaller, present adequate diagnostic information [13, 15, 19]. Using specific indication-dependent optimization in case of tooth auto-transplantation in children, sufficient information from CBCT-examination has been achieved with FOV size 5 × 5 cm [13]. For impacted maxillary canines, minimum FOV size with optimal positioning could be smaller than standard ones, 4 × 3.5 cm [19]. When comparing three different FOV sizes (3 × 4 cm, 4 × 4 cm and 6 × 6 cm) the largest FOV presented the lowest diagnostic level for implant planning, possibly due to scattered radiation, and, in contrast, the smallest FOV size the lowest diagnostic level for periapical diagnosis, 4 × 4 cm obtaining the best overall ranking [15].

As a main result of our study, minimum volumes that would cover all lower third molars and the MC, excluding pathological findings, would be 2.6∅ × 2.8 cm on the right side, and 3.2∅ × 3.2 cm on the left. Completed root length development of all lower third molars of our patients ensured that the FOV was not underestimated, and representation of a large variety of third-molar inclinations increases the generalizability of the results. Because measurements of the tooth position displayed no significant differences between the right and left side, our recommended FOV for either side in practice is 3.5∅ × 3.5 cm. This volume size encompasses the object and 2–3 mm of surrounding bone that, based on the listed criteria of a good periapical radiograph, should be visible in the imaging field for an assessment of adequate periapical anatomy [28]. In addition, proper FOV placement, as documented in the present study, ensures sufficient visibility of the distal part of the second molar to, importantly, diagnose second molar root resorption or distal bone loss.

Principally, imaging of lower third molars with pathological findings requires larger volumes. Here, their inclusion increased the required FOV up to 5.1∅ × 3.9 cm, due to suspected dentigerous cysts that occurred more frequently on the left. When estimating necessity of CBCT-scanning and required FOV size, a decision should be based on the preoperative DPT. Primarily, minimum FOV size should be used, but a larger FOV is justified when it gives additional information for diagnostics of pathological changes or treatment planning.

The other study objective was to define the optimal positioning of FOV. The minimum FOV is a small volume, and its proper positioning is diagnostically vital. All chosen landmarks were based on anatomical structures of the lower second molar, which, unless extracted or heavily distorted, should serve a solid reference for cylindrical FOV placement. Anatomical reference points can be exploited in scout views. Scout view plays an important role in selection and positioning of FOV. In CBCT imaging, there is no computer-aided connecting factor between a scout view and adequate FOV positioning. In medical CT, automatized centering technique, based on the scout view, has been documented to help in optimum patient centering and decrease the radiation dose [20], and studies have been made on additional, low-dose 3D scout scans [29]. A goal is similar development in dental CBCT-imaging: possibility to utilize 2D-scout views for automatic FOV placement and fixation of eventual errors before the final exposure [19].

A limitation of the study is a relatively small study sample. By increasing the number of cases, the resulting compound FOV volumes would likely slightly increase, and right-left differences decrease, since pathological findings associated with lower third molars have been recorded to show bilateral similarity [30]. An assumption of increased resultant imaging volume after examining a larger number of study scans is opposed, however, by the clinical fact that there is seldom an indication for third molar removal if it has not perforated the marginal bone cortex, diminishing the likelihood of a more widespread locational variability among teeth to be scanned preoperatively. Previous 3D assessment studies of teeth or dental follicle dimensions from CBCT scans have had similar sample sizes [19, 31]. A future goal, requiring analysis of extended material, would be next-level optimization of the FOV and its placement according to the DPT view, regarding both impacted third molars and canines.

Many CBCT users perform CBCT-scanning with pre-set imaging programs instead of optimizing the imaging parameters [9]. Knowing the minimum FOV size with optimal indication-specific positioning offers a theoretical base for development of indication-specific preprograms for dental imaging. CBCT-preprograms with automatized FOV placement would facilitate proper placing and reduce possible errors. Proper minimum FOV placement and lowering radiation exposure of CBCT-examination would promote replacement of a conventional 2D radiograph by a diagnostically more accurate 3D CBCT-scan. This requires, however, further studies of different indication-specific FOV-positions and cooperation with manufacturers. On the other hand, the present study results are independent of used CBCT-device type or manufacturer and take a step towards device-independent international standards of CBCT-optimization.

## Conclusions

Indication-specific optimization is necessary in terms of improvement of patient radiation protection in CBCT-examinations. FOV volume 3.5∅ × 3.5 cm would cover all right- and left-side lower third molars and MC if proper positioning is possible and there are no associated pathological findings. The optimized FOV is diagnostic also regarding eventual second molar root resorption or distal bone loss. FOV volume 5.5∅ × 4.0 would also encompass the pathological findings associated with third molars in the present study, but case-specific adjustment is recommended based on the preoperative DPT.

## Data Availability

The datasets generated and analyzed during the study are not publicly available due to patient privacy, but are available from the corresponding author upon reasonable request.

## Abbreviations

CBCT: Cone-beam computed tomography
FOV: Field-of-view
MC: Mandibular canal
DPT: Dental panoramic tomography
2D: Two-dimensional
3D: Three-dimensional
CT: Computed tomography
MME: Method of moment’ estimator
DAP: Dose area product
